# Optimizing intersectoral collaboration and citizen participation in community-level health promotion: a scoping review

**DOI:** 10.3389/phrs.2026.1608894

**Published:** 2026-05-26

**Authors:** Jannemiek Sonneveld, Lilian van der Ven, Janna Bruijning, Niek de Wit, Katarina Jerković-Ćosić

**Affiliations:** 1 HU University of Applied Sciences, Research Group Innovation in Preventive Healthcare, Utrecht, Netherlands; 2 Julius Center for Health Sciences and Primary Care, University Medical Center Utrecht, Utrecht, Netherlands; 3 Public Health and Oral Care Department, Academic Centre for Dentistry Amsterdam (ACTA), VU University, Amsterdam, Netherlands

**Keywords:** citizen participation, community-based, health promotion, intersectoral collaboration, scoping review

## Abstract

**Objectives:**

We aimed to map facilitators and barriers for both intersectoral collaboration (IC) and citizen participation (CP) in community-level health promotion.

**Methods:**

In this scoping review, we searched PubMed, Embase, PsycInfo, CINAHL, Scopus, Global Health, Sociological Abstracts, and Social Services Abstracts, using keywords pertaining to IC, CP, health promotion, and local context to identify studies published between January 2013 and March 2025.

**Results:**

We included 44 studies: 16 on IC, 16 on CP, and 12 on both. Facilitators and barriers can be divided into six overarching themes: Shared purpose, Operationalization, Relational dynamics, Adequate structure and support, Essential people at the table, and Personal drives. The frequency and interpretation of these themes differed between IC and CP.

**Conclusion:**

Facilitators and barriers for both IC and CP in community-based health promotion can be divided into six themes, with both similarities and distinguishing differences existing between IC and CP. Our study presents a framework that reflects the realities of collaboration across sectors and with citizens, which can be used to initiate, sustain, evaluate, and optimize health promotion collaboration processes.

## Introduction

Global life expectancy at birth has increased from 50,9 years in 1960 to 73,3 years in 2023 [1]. Creating serious challenges for healthcare systems worldwide. First, higher life expectancies lead to increased prevalence of chronic diseases and multimorbidity [2] and rising healthcare consumption and costs. A second challenge for healthcare systems is staff shortages. As the working population is expected to increase only slightly or possibly decrease over the next 20 years, the influx of new staff into healthcare will be insufficient [3]. Additionally, current healthcare professionals are leaving the field due to high workloads, early retirement, or more attractive opportunities [4]. Lastly, while life expectancy is increasing, so are health disparities. People with higher socioeconomic status have become healthier than those with lower socioeconomic status. People in vulnerable conditions, like loneliness, poverty, unemployment, and stress, are especially at risk of health problems [5].

These challenges have urged many countries to shift their focus towards health promotion [2]. Health promotion activities aim to foster healthy behaviors within a supportive social and physical environment. Such efforts are a core component of public health, which focuses on improving population-level health. Within the broader healthcare system, public health complements other sectors by addressing upstream determinants of health, that are often situated outside the healthcare domain. Its workforce comprises both medical and non-medical professionals. To prevent the specific health problems of a community, it is important to base health promotion activities on local needs; as the Shanghai Declaration states, “health is created at the local level in the setting of everyday life, in the neighborhoods and communities where people of all ages live, love, work, study and play.” [5]. By organizing health promotion at the community-level, the need for care is expected to diminish. Organizing health promotion at the community-level is also thought to stimulate self-management. By strengthening local networks, citizens can use their own networks more often, helping to reduce healthcare consumption and health disparities, and increasing the quality of life [6, 7]. As a wide range of determinants influences health [8] effective community-level health promotion requires intersectoral collaboration (IC) between public health, primary care, and other stakeholders, both at the professional and organizational level [9]. Such collaboration should be based on a broad perspective of health, specifically for people in vulnerable conditions.

It remains unclear how optimal IC can be achieved [10], although various facilitators and barriers have been reported. Facilitators include leadership, communication, coordination, and sharing of data to identify and address underlying causes of health problems [11, 12]. Barriers include a lack of time, shared goals, financial space, and organizational support [13]. Organizational differences also hinder IC; stakeholders have their own priorities, plans, conditions, systems, perspectives, and approaches to promoting the public’s health and wellbeing [14].

Additionally, a lack of citizen participation (CP) is considered a barrier to successful IC and health promotion [15]. CP in developing and evaluating health promotion activities can lead to better alignment with the target group. When interventions and policies are tailored to citizens’ priorities, their efficacy increases [16].

Thus, IC and CP are both important and interwoven prerequisites for successful community-level health promotion. However, previous literature reviews only focused on IC. We found no previous reviews on CP in community-level health promotion, nor reviews that focused on both IC and CP. Furthermore, existing reviews on IC had a strong focus on clinical practice and disease prevention [10, 13], a limited selection of search terms and/or databases [10, 17] and/or included studies limited to specific partnerships [17] or specific countries [13, 18]. The interwovenness of IC and CP, together with the growing body of literature on these topics, warrants a new review, focusing on both topics, with the aim of mapping and comparing facilitators and barriers for IC and CP in community-level health promotion. This overview integrates insights into the drivers of collaboration and the mechanisms for participation within a community and supports the public health workforce at various levels.

## Methods

We conducted a scoping review to map facilitators and barriers related to IC and CP in community-level health promotion. As these are broad concepts and the evidence base is heterogeneous in design and in sectoral context, a scoping approach is suitable for mapping and synthesizing themes [19]. In conducting our review, we followed the Johanna Briggs Institute guidance for scoping reviews and the PRISMA Extension for Scoping Reviews guidelines [20, 21]. Our study protocol was registered prospectively on the Open Science Framework on 14 March 2023 (file number j6nc3).

### Eligibility criteria

We formulated our content-related eligibility criteria according to the Participants–Concept–Context (PCC) mnemonic, recommended for scoping reviews by the Joanna Briggs Institute [21]. Participants were defined as professionals working in health promotion – either in public health, primary care, or other sectors – and citizens or communities. The first concept we defined was IC, which was considered as collaboration on health promotion between professionals from two or more sectors, as defined under Participants. The second concept was CP, which we considered as the involvement of citizens and communities in developing health promotion interventions or policies. Health promotion was defined as interventions and policies that aim to foster healthy behaviors within a supportive social and physical environment. The context was defined as local settings such as a neighborhood, community, or district. Studies were eligible if they mentioned facilitators and barriers for IC and/or CP. Only original research published in English or Dutch in 2012 or later was included. Although our protocol did not explicitly exclude non-empirical publications, full-text screening revealed a substantial volume and diversity of empirical studies. We estimated that non-empirical publications would have limited added value and decided to include only original research. The date range was selected to ensure relevance to current policy and practice, and to balance comprehensiveness with feasibility. A detailed overview of the inclusion and exclusion criteria is listed in Supplementary Appendix I.

### Search strategy

We employed a three-step search strategy. First, we conducted a limited search in PubMed and Embase to identify highly relevant articles or so-called “key papers.” In these papers, we identified keywords in title, abstract, and index terms. Second, we conducted a search in PubMed, Embase, PsycInfo, CINAHL, Scopus, Global Health, Sociological Abstracts, and Social Services Abstracts, using the keywords and index terms identified in step 1. The search string for this search was built in two parts. The first part, about IC, consisted of four sets of search terms. The second part, about CP, consisted of three sets of search terms. Both parts were based on the PCC-mnemonic. The two parts of the search strategy were combined using OR. The search terms are listed in Table 1, the complete search strings for all databases are presented in Supplementary Appendix II. The search was conducted on 23 March 2023. A search update was conducted on 19 February 2025. Lastly, after study selection, we screened the reference lists of all included full-text articles for additional studies.

**TABLE 1:** Search terms for IC (set A) and CP (set B) in community-level health promotion (intersectoral collaboration and citizen participation, scoping review, global, 2012–2025).

Set A	Category	Search term
S1 AND	Participant	“Public health” AND (“primary healthcare” OR “primary care” OR “primary healthcare”)
S2 AND	Concept	Collaborat* OR cooperat* OR integrat* OR cross-sectoral OR transdisciplinary OR multidisciplinary OR partnership OR interdisciplinary OR interprofessional OR intersectoral OR “intersectoral collaboration”
S3 OR	Concept	“Health promotion*” OR “preventive service*” OR “community health” OR “population health” OR “preventive health service*” OR “preventive healthcare” OR “preventive health program*” OR “preventive program*”
S4	Context	Local OR district OR community OR neighbourhood OR neighborhood OR “population-based”

Set B	Category	Search term
S1 AND	Participants	Citizen OR citizens OR “citizen participation” OR “citizen science” OR “community engagement” OR “community participation” OR “community action*” OR “community involvement*”
S2 AND	Concept	“Health promotion*” OR “preventive service*” OR “community health” OR “population health” OR “preventive health service*” OR “preventive healthcare” OR “preventive health program*” OR “preventive program*”
S3	Context	Local OR district OR community OR neighbourhood OR neighborhood OR “population-based”

### Study selection

The search results were uploaded to Zotero 6.0.36, and duplicate records were removed. Inter-rater reliability sessions were conducted to ensure consistency between reviewers. Three reviewers (JS, LV, and JB) independently reviewed 25 records, after which they discussed discrepancies until consensus was reached. This process was repeated with two reviewers (JS and LV), with the third reviewer (JB) being consulted when consensus could not be reached.

The two reviewers then conducted title and abstract screening. For this process, we used ASReview 1.5, an open-source tool that applies active machine learning to continuously prioritize relevant records based on previous screening decisions. We followed the SAFE procedure as described by Boetje and Van de Schoot [22], which consists of four phases: Screen, Apply, Find, and Evaluate.

In the Screen phase, 1% of records were screened and labeled as “relevant” or “irrelevant” to train the active learning model. Based on the results of the Screen phase, the number of relevant records in the total dataset was estimated.

During the Apply phase, the two reviewers independently screened the dataset. Instead of following the stopping criterion described in our protocol – no relevant records identified in the last 100 records – we adopted the stopping criteria described in the SAFE procedure. This means we screened the dataset until the following four stopping criteria were met:

1. All key papers were labeled as relevant.
2. At least twice the estimated number of relevant records had been screened.
3. A minimum of 10% of the total dataset had been screened.
4. No relevant records had been identified in the last 50 records.

In cases of doubt, the full-text article was reviewed to ensure that the active learning model was adequately trained.

During the Find phase, a deep learning model was employed to ensure that no relevant records were missed. The labeling decisions from the Screen phase were used as prior knowledge to train the deep learning model, which then re-ordered the remaining unlabeled records. The reviewers then continued screening until no extra relevant records were identified in the last 50 records. After the Find phase, the reviewers compared their independently labeled records. Discrepancies were discussed, with the third reviewer consulted when consensus could not be reached.

The datasets of both reviewers were then combined into one file, with which the Evaluation phase was conducted. The active learning model for this phase was trained using the 10 highest- and lowest-ranked records from the Find phase. The third reviewer then screened the previously labeled records to identify any relevant ones that had been excluded. Screening was stopped when no extra relevant records were identified in the last 50 records.

For the updated search conducted in February 2025, one reviewer (JS) screened new titles and abstracts in ASReview. All full-text articles included in the initial search were used as prior knowledge. Screening was stopped when no extra relevant records were identified in the last 50 records.

Full-text articles were uploaded and screened using Zotero. The two reviewers independently screened all articles. Disagreements about inclusion were resolved through discussion, and when necessary, the third reviewer was consulted. Finally, the reference lists of all included articles were screened to identify additional relevant studies.

### Data charting and analysis

We charted data from all included studies using the data charting form presented in Supplementary Appendix III. This is a revised version of the form presented in the study protocol, as we identified excessive overlap between categories. We charted data on study characteristics, facilitators, and barriers. After data charting, we conducted a thematic analysis of facilitators and barriers for IC and CP using NVivo 14 [23]. Text related to facilitators and barriers was coded and discussed iteratively by two researchers (JS and LV) to further refine the codes. Then, the two researchers reviewed the codes and their relationships, leading to the creation of categories and themes.

## Results

### Study selection

**FIGURE 1:**
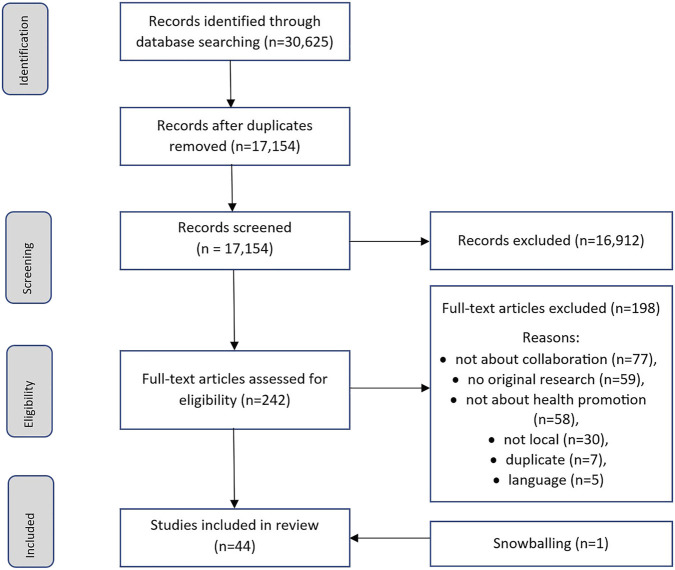
Flow chart of the study selection process (intersectoral collaboration and citizen participation, scoping review, global, 2012–2025).

A flow chart of the study selection process is presented in Figure 1. The numbers in this flow chart are the combined numbers from the initial search and the search update.

The initial search, conducted in March 2023, yielded 23,143 records. After removing duplicates, 12,986 records were imported into ASReview. In the Screen phase, 129 records were screened and labeled as ‘relevant’ or ‘irrelevant’ to train the active learning model. Based on the results of the Screen phase, the estimated number of relevant records in the total dataset was 2,059, indicating that a minimum of 4,118 records needed to be screened in the Apply phase to meet stopping criterion 2. Title and abstract screening using the SAFE procedure yielded 239 articles for full-text screening. After full-text screening, 41 studies were included in our review. The search update in February 2025 resulted in 22 additional potentially relevant records after title and abstract screening, of which three were included after full-text screening, yielding a total of 44 included studies.

### Study characteristics

Table 2 presents an overall summary of the study characteristics. The characteristics of the individual studies are provided in Supplementary Appendix IV. The included studies were published between 2012 and 2025, with the majority appearing between 2018 and 2020. In most studies, the focus of health promotion was healthy weight, food, and/or obesity (n = 26). Most studies (n = 21) employed mixed methods or a qualitative design (n = 18).

**TABLE 2:** Summary of study characteristics (intersectoral collaboration and citizen participation, scoping review, global, 2012–2025).

Study characteristics	Categories	N (%)
Year of publication	2012–2014 2015–2017 2018–2020 2021–2025 (February)	6 (14%) 11 (25%) 17 (39%) 10 (23%)
Country of origin of lead author (institution)	North America Europe Oceania Asia	23 (52%) 14 (32%) 6 (14%) 1 (2%)
Focus of health promotion^*^	Healthy weight/Food/Obesity Physical activity Mental health Overall (community) health Social wellbeing Smoking and/or alcohol Cardiovascular disease Participation in primary healthcare Integration of PC and PH	26 (59%) 11 (25%) 7 (16%) 4 (9%) 4 (9%) 2 (3%) 3 (7%) 1 (2%) 1(2%)
Study design	Mixed methods Qualitative Community-based participatory research	21 (48%) 18 (41%) 5 (11%)

PC, primary care; PH, public health.

* A study can focus on more than one topic.

### Synthesis of results

**FIGURE 2:**
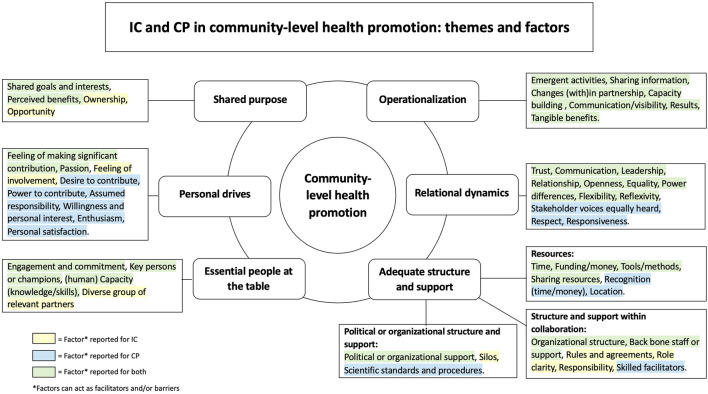
Framework of themes and factors in IC and CP in community-level health promotion (intersectoral collaboration and citizen participation, scoping review, global, 2012–2025).

We summarized the results of our data charting and analysis in accordance with the study objective: to map facilitators and barriers for IC and CP in community-level health promotion. The results are presented as a narrative summary, supported by an illustrative framework (Figure 2).

Our main findings are organized into six themes. For each theme, we describe the factors it encompasses. These factors can act as facilitators, barriers, or both, and they relate to IC, CP, or both. When factors apply to both IC and CP, their specific interpretation may vary across these contexts. Table 3 provides an overview of themes, facilitators (F), and barriers (B) for IC and CP in the included studies.

**TABLE 3:** Overview of themes, facilitators, and barriers for IC and CP in the included studies (intersectoral collaboration and citizen participation, scoping review, global, 2012–2025).

Study	IC								CP							
​	Shared purpose	Operationalization	Relational dynamics	AS&S - resources	AS&S - structure and support within collaboration	AS&S - political or organizational structure and support	Essential people at the table	Personal drives	Shared purpose	Operationalization	Relational dynamics	AS&S - resources	AS&S - structure and support within collaboration	AS&S - political or organizational structure and support	Essential people at the table	Personal drives
Chaisson [24]	F/B	F	F/B	F	F	F	F/B									
Cheadle [25]	F	F	F	F	F		F/B									
De Jong [26]	F	F	F	F/B	F	F/B	F/B									
Hargreaves [27]	F		F/B	F/B	F		F/B									
Holt [28]	F/B	F	F	F	B											
Igel [29]	F/B	F	F/B	F/B	F	F/B	F/B									
Jones [30]	F		F	F	F		F									
Kennedy [31]	F	F	F	F			F									
Lachance [32]	F/B	F/B	F/B	F/B	F/B	F/B	F/B									
Matheson [33]	F/B	F/B	F/B	F/B	F/B	B	F/B									
McNeish [34]	F	F	F/B	F/B	F/B		F/B									
Middleton [35]	F/B	F	F/B	F/B	F/B		B									
Pratt [36]	F	F	F/B	F/B	F/B	F	F	F								
Sjögren Forss [37]	F/B	F	F/B	B	F	B	F/B	F								
Storm [14]	F	F	F	F/B	F/B	F/B	F/B	F								
Toft [38]	F/B	F	F/B	F/B	B	F/B										
Agénor [39]											F/B	B	F/B	B	F/B	
Curbach [40]											F/B	F	B		F/B	B
Cutforth [41]									F	F	F	F/B	F		F/B	B
Davis [42]									F/B	F/B	F/B	F/B	F/B	F/B	F/B	F/B
De Marco [43]											F	F/B	F		F/B	
Derose [44]											F	F	F		F	
Dodgen [45]											F	F	F		F	
English [46]										F/B	F/B	F		B	F	
Fialkowski [47]									F		F/B	F	F	B	F	F
García-Rivera [48]										F/B	F/B	F/B	F	F/B	F/B	B
Korn [49]									F	F	F	F	F		F	F
Lolacono Merves [50]									B	F/B	F/B	F/B	F/B		F/B	B
Majee [51]									B	F	F/B	F/B	F	B	F	
Ochieng [52]									F		F/B	F				F
Quinn [53]									F	F	F	F			F	
Vargas [54]									F		F/B	F			F	
Corsino [55]	F/B		F/B	F	F		F		F	F	F/B	F/B	F		F	B
De Jong [56]	F	F/B	F	F	F/B	B	F		F		F	F/B	F		F/B	
Heo [57]	F/B		F/B	F/B	F/B	B	B		B		F/B				F/B	
Hilgendorf [58]	F/B	F	F/B	F	F		F/B		F	F	F	F	F		F	
Jenkins [59]	B	F	F/B	F/B	F/B	F/B	F/B		F		F/B	F/B	F/B	F	F/B	B
Johnson-Shelton [60]	F	F/B	F	F/B	F		F		F	F	F	F	F	B	F	F
McEvoy [61]						B	F		B	F	F/B	F/B	B	F/B	F/B	F
Nelson [62]	F/B	B	F/B	F	F		F/B				F	F				
Ottesen [63]			F/B	B	F	B	B				F/B	B	F	B	B	
Powell [64]		F/B	F/B		F/B		F		F/B		F	F	F	F	F/B	
Rämgård [65]	F	F	F	F/B	B		F		F	F	F	F			F	
Vermeer [66]	F	F	F	F	F	F	F/B								B	F
n total	25	22	27	26	26	15	26	3	18	13	27	26	20	11	26	13
% total	89%	79%	96%	93%	93%	54%	93%	11%	67%	48%	100%	96%	74%	41%	96%	48%

AS&S = Adequate structure and support; F = theme reported as facilitator; B = theme reported as barrier; F/B = theme reported as facilitator and barrier.

#### Shared purpose

“Shared purpose” concerns factors related to visions, ambitions, and motives for collaboration. Facilitators within this theme for IC are shared goals, priorities, benefits, and perceptions of success. Co-ownership, accommodating interests, and recognizing the collaboration as an opportunity strengthen partnerships. The factors above are described as barriers when they are not met.

In CP, facilitators mostly concern reciprocity in perceived benefits, interests, and mutually beneficial goals by building on the community’s needs and motivations. Facilitating factors are related to citizens and what is relevant to their lives and needs, which helps establish ownership. The absence of shared aims and interests is reported as a barrier.

#### Operationalization

“Operationalization” entails the active process of collaborating and the results achieved. Capacity building, emergent activities, and information sharing facilitate IC, as does the development of skills, knowledge, and capacity of individuals and the coalition as a whole. Achieving results and experiencing tangible benefits contribute to active collaboration. Although some studies report staff changes within partnerships as a facilitator, this is the most commonly reported barrier to operationalizing IC.

Similar findings are found for CP, although the language and terms used to describe facilitators differ. Sharing information and activities, learning together, and exchanging ideas facilitate active collaboration with citizens. The only reported barrier is changes in staff or participants within partnerships.

#### Relational dynamics

“Relational dynamics” refers to factors related to soft skills and the interactions between individuals or groups within a collaboration. Trust and strong relationships facilitate IC. Positive past experiences contribute to building relationships. Mutual communication, dialogue, and reflection enhance trust and encourage progress in the partnership. On the other hand, miscommunication, lack of communication, and leadership issues decrease trust and cohesion.

Equality, acknowledgment, being heard, openness, and respect are reported as facilitators for CP. Investing in relationships and building trust are essential, as are leadership, flexibility, responsiveness, and following the participant’s pace. Communication is mainly reported as a barrier, in the form of a lack of communication, or miscommunication due to the use of language unsuitable for the community. Also, lack of reciprocity, inequality, power differences, and the perception of being used are barriers. Lastly, lack of trust and weak relationships hinder participation.

#### Adequate structure and support

“Adequate structure and support” relates to organizational structures, conditions, and support that are needed on different levels. It is divided into three subthemes: Resources, Structure and support within collaboration, and Political or organizational support.

##### Resources

“Resources” include time, money, tools, and methods. Investing sufficient time in collaboration and joint decision-making is reported as a facilitator for IC. In addition, (sustainable) financing and sharing of resources are helpful. Also described is the importance of using appropriate tools, methods, and participatory approaches for joint decision-making and identifying community assets. Methods mentioned are asset-based community development, community-based participatory research, co-creation, and community assessment. The most commonly reported barriers are lack of time, money, and people.

For CP, resource investment to build skills and relationships is explicitly mentioned, as is respecting the time citizens invest by compensating them and organizing meetings at convenient times and locations. Time for social interaction and a welcoming space facilitate participation. As in IC, the design of meetings and activities is important: adding an educational component contributes to community knowledge and capacity building. Community resources, such as local knowledge and access to assets, are seen as facilitators that should be recognized. The main barriers are lack of time and money. Competing professional and personal demands decrease participation.

##### Structure and support within collaboration

“Structure and support” refers to how the collaboration itself is organized. Facilitating factors are related to clarity, including a clear definition and division of roles and responsibilities, a clear organizational structure, and rules and agreements, such as a clear decision-making process. The availability of backbone staff to organize meetings and facilitate group processes also promotes collaboration. The absence of these factors hinders collaboration, as does the presence of a highly hierarchical organizational structure.

CP is facilitated by the presence of adequate support, backbone staff, and skilled facilitators to ensure that citizens can participate as true partners. It is hindered by difficulties in scheduling meetings at suitable times and places.

##### Political or organizational structure and support

“Political and organizational structure and support” entails factors related to how organizations involved in collaborations are organized themselves, and to the commitment and backing from these organizations and from policymakers. Buy-in from decision-makers helps to allocate resources, facilitate employees, and secure long-term participation. Lack of support and top-down decision-making result in resistance. Organizational and political silos inhibit action and shared goals.

Specifically in research partnerships between academia and citizens, scientific standard and procedures can be a facilitator when research and community needs are balanced. In the absence of flexibility and when there is an imbalance between research activities and the needs of the community, tension and frustration can occur and hinder participation. The pace of academic research processes, and the occurrence of paralysis by analysis, can conflict with the urgency or preferred speed of action within the community.

#### Essential people at the table

“Essential people at the table” concern factors related to the active presence of relevant stakeholders based on role, expertise, and skills. It relates to the identification of ‘the right people’ and their being present, in the right role. Having a diverse group of stakeholders facilitates collaboration, as does the presence of champions, who play a crucial role in building and sustaining partnerships. However, merely being present is not enough; another important facilitator is participants’ active and sustained involvement, engagement, and commitment. Barriers to IC are limited diversity of participants or losing participants because of competing priorities.

In CP, connecting to existing community groups or initiatives helps to find the right people. Commitment and engagement are key facilitators here, either by involving intrinsically motivated people or by actively stimulating commitment and engagement. The presence of key persons and champions is also a facilitator for CP, for instance, due to their knowledge and expertise of the community. Barriers are seen in the inability to sustain participation and engagement.

#### Personal drives

“Personal drives” entails a person’s enthusiasm, passion, and motivation to be involved. In IC, passion, the feeling of involvement, and the spirit to change things together and make a difference are reported as facilitators. However, personal drives are mainly reported for CP. Participants have the desire to contribute, the feeling of making a significant contribution, the power to contribute, and an assumed community responsibility. Positive experiences and personal satisfaction keep citizens active. On the other hand, in the absence of willingness and personal interest, it is challenging to maintain the community’s enthusiasm.

### Comparison of IC and CP

**FIGURE 3:**
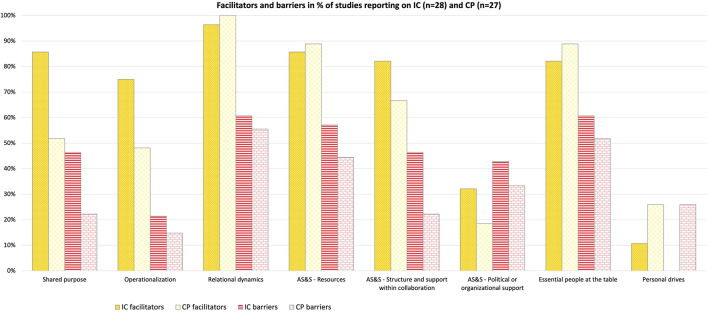
Facilitators and barriers in % of studies reporting on IC and CP (intersectoral collaboration and citizen participation, scoping review, global, 2012–2025).

Of the 44 studies we included, 16 reported facilitators and/or barriers for IC, 16 reported facilitators and/or barriers for CP, and 12 reported both. Overall, facilitators are reported more often than barriers. Relational dynamics, Essential people at the table, and Resources are the most frequently reported themes for IC (96%, 93%, and 93%, respectively) and for CP (100%, 96%, and 96%). Shared purpose and Operationalization are reported more frequently for IC (89% and 79%) than for CP (67% and 48%). Personal drives are reported more frequently for CP (48%) than for IC (11%). Figure 3 provides a visual comparison of reported themes in facilitators and barriers for IC and CP.

## Discussion

The aim of this scoping review was to map facilitators and barriers for IC and CP in community-level health promotion. Based on 44 included studies, we identified 6 overarching themes for facilitators and barriers: Shared purpose, Operationalization, Relational dynamics, Adequate structure and support, Essential people at the table, and Personal drives.

### Comparing facilitators and barriers for IC and CP

#### Similarities

Both in IC and in CP, the most frequently reported themes were Relational dynamics, Essential people at the table, and Resources. Investing time to build trusting relationships fosters sustained involvement, and commitment. Time, financing, and information sharing – along with appropriate tools, methods and thoughtful design of meetings contribute to capacity building. Political and organizational support and a sound organizational structure, facilitates collaboration. Shared goals that benefit participating organizations and the community help maintain motivation and reinforce a meaningful contribution.

#### Differences

For IC, essential factors are ownership, rules and agreements, and role clarity. Having a diverse group of relevant partners, including key persons or champions, is important. Attention should be paid to silos in organizational structures that hinder collaboration.

For CP, the importance of personal drives is emphasized more. Citizens are motivated by personal interest, willingness, enthusiasm, and the desire and power to contribute. They feel responsible for having the voices of their community heard.

### Comparison with previous research and frameworks

Overall, the six themes we identified were similar to previous reviews on IC [10, 12, 13, 17, 18], with one exception: no previous reviews report on the personal drives of participants. A possible explanation for this is that these reviews focused on IC alone, while the personal drives in our scoping review were mainly related to CP. At the factor level, communication, leadership, financing, organizational support, and shared goals are commonly identified. Factors not often identified are engagement, organizational benefits, and the visibility of results; these are only described by Leenaars et al [17]. Attention to tools, methods, and the design of meetings and activities to contribute to capacity building are reported by Rechel [10] and Martin-Misener et al [13]. Rechel [10] included the review by Martin-Misener et al [13], which explains their similarity in reported factors.

The importance of community engagement is described in three of the reviews on IC [10, 13, 18]. However, we found no previous reviews on CP in health promotion. CP is often defined as participation in health promotion activities, not in the development of activities or policies.

Visualizing our results in a framework proved valuable for interpreting the outcomes of our study. When comparing our framework to existing models, we found substantial resemblance with the Center for Community Health and Evaluation (CCHE) collaborative model [25, 67]. Other relevant models were less applicable. For example, both the HALL framework [68] and the framework by San Martín-Rodríguez et al. [69] – which guided the review by Martin-Misener et al. [13] – categorize determinants of collaboration across three levels: systemic/institutional, organizational, and interactional/interpersonal. This classification enables a structured analysis of the multi-level dynamics that influence collaboration. However, we identified several dimensions that are underrepresented or absent in these original models compared to our findings, for example, the presence of essential people at the table and the role of personal drives. The latter may be explained by the fact that these models focus on collaboration between primary care and public health, not on integrating CP into IC. We did find this critical role of stakeholder inclusivity in the CCHE model, which places the community and equity at the center of collaboration.

Although our themes broadly align with those in the CCHE model, several distinctions emerged. First, we identified “Personal drives” as a separate theme to capture intrinsic motivation for collaboration. Second, we split “Adequate structure and support” into three subthemes to more precisely locate facilitators and barriers. Finally, we incorporated leadership within the broader theme of “Relational dynamics,” emphasizing the relational soft skills that underpin effective collaboration.

### Limitations

Several limitations of our scoping review should be acknowledged. Firstly, we found that the definitions of concepts of interest to our review, such as health promotion, CP, and public health, varied considerably between studies, or it was unclear how these concepts were defined in the studies. This made it challenging to decide whether a study met our eligibility criteria, possibly resulting in the omission of relevant studies. For example, in some cases, it was difficult to determine whether CP indicated participation in the development of health promotion policies and activities, in the implementation of these activities, or participation in the activities themselves.

Secondly, we used ASReview for the title and abstract screening. This proved valuable for efficiently managing the large number of records our search yielded, as it significantly reduced the number of records that needed to be screened. However, a consequence of this approach is that we did not screen all titles and abstracts, which may have resulted in the omission of relevant studies.

Finally, it is worth noting that this review was designed to map facilitators and barriers for IC and CP; exploring how these facilitators and barriers relate to each other was beyond its scope.

### Practical relevance

Our findings and framework can support policymakers and stakeholders at various levels of the public health workforce seeking insight into the factors that influence IC and CP, as well as how to initiate, sustain, evaluate, and optimize collaboration processes.

At the professional level, when initiating a community-level health promotion collaboration, it is essential to involve all relevant stakeholders from the outset, including citizens. Establish a shared vision and common goals collectively to ensure that the interests of the various partners and the needs of the community are met. Meaningful CP requires treating citizens as equal partners, actively listening to their concerns and priorities, and fostering the exchange of diverse perspectives. This not only enhances the relevance of results but also contributes to building mutual trust. Participants’ differing motivations need to be recognized as well. Professionals typically participate on behalf of their organizations within a formalized structure governed by rules and agreements. In contrast, residents are personally invested, as the collaboration concerns their living environment and their daily lives.

At the organizational level, roganizational support is a key factor: IC can be challenging and is unlikely to succeed without adequate support in terms of time, money, commitment, and ownership.

In general, allowing sufficient time for the transition to a collaborative working structure is essential to foster sustainable and effective partnerships.

### Conclusions

With our scoping review, we mapped six overarching themes concerning facilitators and barriers for IC and CP in community-level health promotion: Shared purpose, Operationalization, Relational dynamics, Adequate structure and support, Essential people at the table, and Personal drives. Understanding the drivers and mechanisms of IC and CP can support evidence-informed decisions at various levels of public health collaboration. Our framework can serve as a tool for evaluating and improving the diverse aspects of the IC and CP processes.
