# Supplementary material for: Optimizing intersectoral collaboration and citizen participation in community-level health promotion: a scoping review
Source: Public Health Rev. 2026 May 26;47:1608894. doi: 10.3389/phrs.2026.1608894 (PMC13329247; doi:10.3389/phrs.2026.1608894)
Supplement: Supplementary file 1 [file Supplementaryfile1.docx]

Appendices

**Appendix I: Inclusion and exclusion criteria**

| Aspect | Inclusion Criteria | Exclusion Criteria |
| --- | --- | --- |
| Participants | - Professionals working in public health, primary (health) care, or related sectors, and/or - citizens living in the specific neighbourhood or community. | - Professionals working in other non-related sectors |
| Concept | - (intersectoral) collaboration in population-based health promotion, and/or - citizen participation in population-based health promotion | - Disease prevention - Living environment - Monodisciplinary collaboration |
| Context | - Local settings such as a neighbourhood, community, or district | - Larger settings such as town, regional, or state |
| Type of studies | - Original research from 2012 - Studies published in English and Dutch | - Reviews - Conference abstracts and editorials - Non-empirical studies and grey literature - Studies published in other languages |

**Appendix II: Search strategy databases**

For the updated search, we used the original search string and replaced the date range filter with 2023 to the present. For the database Global Health, we searched in Descriptor, Broad term, Identifier, and Indexing term, since searching in Cabicode was not possible anymore.

| Database/Search Engine | Search Strategy |
| --- | --- |
| PubMed | (((local[tiab] OR district[tiab] OR communit*[tiab] OR neighbourhood[tiab] OR neighborhood[tiab] OR "population-based"[tiab] OR "health promotion*"[tiab] OR "preventive service*"[tiab] OR "population health"[tiab] OR "preventive health service*"[tiab] OR "preventive health care"[tiab] OR "preventive health program*"[tiab] OR "preventive program*"[tiab] OR "health promotion"[Mesh] OR "population health"[Mesh:NoExp]) AND (collaborat*[tiab] OR cooperat*[tiab] OR integrat*[tiab] OR cross-sectoral[tiab] OR transdisciplinary[tiab] OR multidisciplinary[tiab] OR partnership[tiab] OR interdisciplinary[tiab] OR interprofessional[tiab] OR intersectoral[tiab] OR "intersectoral collaboration"[Mesh]) AND ("public health"[tiab] OR "public health"[Mesh]) AND ("primary health care"[tiab] OR "primary health care"[Mesh:NoExp] OR "primary care"[tiab] OR "primary healthcare"[tiab])) OR ((citizen [tiab] OR citizens [tiab] OR "community engagement"[tiab] OR "community participation"[tiab] OR "community action*"[tiab] OR "community involvement*"[tiab] OR "citizen science"[Mesh] OR "community participation"[Mesh]) AND ("health promotion*"[tiab] OR "preventive service*"[tiab] OR "community health"[tiab] OR "population health"[tiab] OR "preventive health service*"[tiab] OR "preventive health care"[tiab] OR "preventive health program*"[tiab] OR "preventive program*"[tiab] OR "health promotion"[Mesh] OR "population health"[Mesh:NoExp]) AND (local[tiab] OR district[tiab] OR communit*[tiab] OR neighbourhood[tiab] OR neighborhood[tiab] OR "population-based"[tiab]))) AND ("2012" : "3000"[DP]) |
| Embase | ((((local OR district OR communit* OR neighbourhood OR neighborhood OR 'population-based' OR 'health promotion*' OR 'preventive service*' OR 'population health' OR 'preventive health service*' OR 'preventive health care' OR 'preventive health program*' OR 'preventive program*'):ti,ab,kw OR 'health promotion'/exp OR 'population health'/exp) AND ((collaborat* OR cooperat* OR integrat* OR cross-sectoral OR transdisciplinary OR multidisciplinary OR partnership OR interdisciplinary OR interprofessional OR intersectoral):ti,ab,kw OR 'intersectoral collaboration'/exp) AND ('public health':ti,ab,kw OR 'public health'/exp) AND (('primary health care' OR 'primary care' OR 'primary healthcare'):ti,ab,kw OR 'primary health care'/exp)) OR (((citizen OR citizens OR 'community engagement' OR 'community participation' OR 'community action*' OR 'community involvement*'):ti,ab,kw OR 'citizen science'/exp OR 'community participation'/exp) AND (('health promotion*' OR 'preventive service*' OR 'community health' OR 'population health' OR 'preventive health service*' OR 'preventive health care' OR 'preventive health program*' OR 'preventive program*'):ti,ab,kw OR 'health promotion'/exp OR 'population health'/exp) AND (local OR district OR communit* OR neighbourhood OR neighborhood OR 'population-based'):ti,ab,kw)) AND ([2012-2023]/py) |
| PsycInfo | (((local OR district OR communit* OR neighbourhood OR neighborhood OR "population-based" OR "health promotion*" OR "preventive service*" OR "population health" OR "preventive health service*" OR "preventive health care" OR "preventive health program*" OR "preventive program*").ab,ti,id. OR exp health promotion/ OR exp population health/) AND ((collaborat* OR cooperat* OR integrat* OR cross-sectoral or transdisciplinary OR multidisciplinary OR partnership OR interdisciplinary OR interprofessional OR intersectoral).ab,ti,id. OR exp collaboration/) AND (("public health").ab,ti,id. OR exp public health/) AND (("primary health care" OR "primary care" OR "primary healthcare").ab,ti,id. OR exp primary health care/)) OR (((citizen OR citizens OR "community engagement" OR "community participation" OR "community action*" OR "community involvement*").ab,ti,id. OR exp community involvement/) AND (("health promotion*" OR "preventive service*" OR "community health" OR "population health" OR "preventive health service*" OR "preventive health care" OR "preventive health program*" OR "preventive program*").ab,ti,id. OR exp health promotion/ OR exp population health/) AND ((local OR district OR communit* OR neighbourhood OR neighborhood OR "population-based").ab,ti,id.)) |
| CINAHL | ((local OR district OR communit* OR neighbourhood OR neighborhood OR "population-based" OR "health promotion*" OR "preventive service*" OR "population health" OR "preventive health service*" OR "preventive health care" OR "preventive health program*" OR "preventive program*" OR MH "Health Promotion+" OR MH "Population Health") AND (collaborat* OR cooperat* OR integrat* OR cross-sectoral OR transdisciplinary OR multidisciplinary OR partnership OR interdisciplinary OR interprofessional OR intersectoral OR MH "Collaboration") AND ("public health" OR MH "Public Health+") AND ("primary health care" OR "primary care" OR "primary healthcare" OR MH "Primary Health Care"))) OR (((citizen OR citizens OR "community engagement" OR "community participation" OR "community action*" OR "community involvement*" OR MH "Citizen Science") AND ( "health promotion*" OR "preventive service*" OR "community health" OR "population health" OR "preventive health service*" OR "preventive health care" OR "preventive health program*" OR "preventive program*" OR MH "Health Promotion+" OR MH "Population Health" ) AND (local OR district OR communit* OR neighbourhood OR neighborhood OR "population-based")) AND (PY 2012-3000) |
| Scopus | TITLE-ABS-KEY ( ( local OR district OR communit* OR neighbourhood OR neighborhood OR "population-based" OR "health promotion*" OR "preventive service*" OR "population health" OR "preventive health service*" OR "preventive health care" OR "preventive health program*" OR "preventive program*" ) AND ( collaborat* OR cooperat* OR integrat* OR cross-sectoral OR transdisciplinary OR multidisciplinary OR partnership OR interdisciplinary OR interprofessional OR intersectoral ) AND "public health" AND ( "primary health care" OR "primary care" OR "primary healthcare" ) ) OR TITLE-ABS-KEY ( ( citizen OR citizens OR "community engagement" OR "community participation" OR "community action*" OR "community involvement*" ) AND ( "health promotion*" OR "preventive service*" OR "community health" OR "population health" OR "preventive health service*" OR "preventive health care" OR "preventive health program*" OR "preventive program*" ) AND ( local OR district OR communit* OR neighbourhood OR neighborhood OR "population-based" ) ) AND PUBYEAR > 2011 |
| Global Health | ((title:(local OR district OR communit* OR neighbourhood OR neighborhood OR "population-based" OR "health promotion*" OR "preventive service*" OR "population health" OR "preventive health service*" OR "preventive health care" OR "preventive health program*" OR "preventive program*") OR ab:(local OR district OR community OR neighbourhood OR neighborhood OR "population-based" OR "health promotion*" OR "preventive service*" OR "community health" OR "population health" OR "preventive health service*" OR "preventive health care" OR "preventive health program*" OR "preventive program*") OR subject:(local OR district OR community OR neighbourhood OR neighborhood OR "population-based" OR "health promotion*" OR "preventive service*" OR "community health" OR "population health" OR "preventive health service*" OR "preventive health care" OR "preventive health program*" OR "preventive program*") OR cabicode:("health promotion")) AND (title:(collaborat* OR cooperat* OR integrat* OR cross-sectoral OR transdisciplinary OR multidisciplinary OR partnership OR interdisciplinary OR interprofessional OR intersectoral) OR ab:(collaborat* OR cooperat* OR integrat* OR cross-sectoral OR transdisciplinary OR multidisciplinary OR partnership OR interdisciplinary OR interprofessional OR intersectoral) OR subject:(collaborat* OR cooperat* OR integrat* OR cross-sectoral OR transdisciplinary OR multidisciplinary OR partnership OR interdisciplinary OR interprofessional OR intersectoral)) AND (title:("public health") OR ab:("public health") OR subject:("public health") OR cabicode:("Public Health")) AND (title:("primary health care" OR "primary care" OR "primary healthcare") OR ab:("primary health care" OR "primary care" OR "primary healthcare") OR subject:("primary health care" OR "primary care" OR "primary healthcare") OR cabicode:("primary health care"))) OR ((title:(citizen OR citizens OR "community engagement" OR "community participation" OR "community action*" OR "community involvement*") OR ab:(citizen OR citizens OR "community engagement" OR "community participation" OR "community action*" OR "community involvement*") OR subject:(citizen OR citizens OR "community engagement" OR "community participation" OR "community action*" OR "community involvement*") OR cabicode:("community involvement")) AND (title:("health promotion*" OR "preventive service*" OR "community health" OR "population health" OR "preventive health service*" OR "preventive health care" OR "preventive health program*" OR "preventive program*") OR ab:("health promotion*" OR "preventive service*" OR "community health" OR "population health" OR "preventive health service*" OR "preventive health care" OR "preventive health program*" OR "preventive program*") OR subject:("health promotion*" OR "preventive service*" OR "community health" OR "population health" OR "preventive health service*" OR "preventive health care" OR "preventive health program*" OR "preventive program*") OR cabicode:("Health Promotion"))  AND (title:(local OR district OR communit* OR neighbourhood OR neighborhood OR "population-based") OR ab:(local OR district OR community OR neighbourhood OR neighborhood OR "population-based") OR subject:(local OR district OR community OR neighbourhood OR neighborhood OR "population-based")) AND yr:[2012 TO 2023] |
| Sociological Abstracts incl. Social Services Abstract | (TITLE,ABSTRACT,IF(local OR district OR community OR neighbourhood OR neighborhood OR "population-based" OR "health promotion*" OR "preventive servic*s" OR "community health" OR "population health" OR "preventive health service*" OR "preventive health care" OR "preventive health program*" OR "preventive program*") AND TITLE,ABSTRACT,IF(collaborat* OR cooperat* OR integrat* OR cross-sectoral OR transdisciplinary OR multidisciplinary OR partnership OR interdisciplinary OR interprofessional OR intersectoral) AND (TITLE,ABSTRACT,IF("public health") OR MAINSUBJECT.EXACT("Public Health")) AND (TITLE,ABSTRACT,IF("primary health care" OR "primary care" OR "primary healthcare") OR MAINSUBJECT.EXACT.EXPLODE("Primary Health Care"))) OR ((TITLE,ABSTRACT,IF(citizen OR citizens OR "community engagement" OR "community participation" OR "community action*" OR "community involvement*") OR MAINSUBJECT.EXACT.EXPLODE("Community Involvement") OR MAINSUBJECT.EXACT.EXPLODE("Citizen Participation")) AND TITLE,ABSTRACT,IF("health promotion*" OR "preventive service*" OR "community health" OR "population health" OR "preventive health service*" OR "preventive health care" OR "preventive health program*" OR "preventive program*") AND TITLE,ABSTRACT,IF(local OR district OR community OR neighbourhood OR neighborhood OR "population-based")) |

**Appendix III: Data charting form**

| Title | Author(s) | Year of publication | Country of origin | Participants | Study design | Context | Facilitators | Barriers |
| --- | --- | --- | --- | --- | --- | --- | --- | --- |
|  |  |  |  |  |  |  |  |  |

**Appendix IV: Characteristics of studies**

| Number | Author, year, Country of origin | Methods of data collection | Focus of the study |
| --- | --- | --- | --- |
| 1 | Agénor (2018), USA[39] | Focus group interviews, CBPR and observations | The Strong Women Strong Girls (SWSG) program, which serves a racially and ethnically diverse population of preadolescent girls, predominantly girls of color in underserved communities of Boston and Pittsburgh. The program aims to empower these girls through positive mentoring relationships with college women, utilizing a curriculum centered on female role models. The partnership with researchers aims to better understand and promote the mental health of these girls, addressing specific social stressors related to gender and race/ethnicity that negatively impact their mental health. |
| 2 | Chaisson (2022), Canada[24] | Focus group interviews | The Alberta Healthy Communities Approach (AHCA), which aims to foster collaboration and partnership within local communities. The goal is to build capacity for community-driven initiatives that address the proximal determinants of health related to cancer and chronic disease prevention. This includes promoting supportive policies and environments that encourage physical activity, healthy eating, UVR protection, and cessation of tobacco and alcohol use, while also being adaptable to community-led actions addressing other social determinants of health. |
| 3 | Cheadle (2019), USA[25] | Document review and interviews | The piloting of accountable communities of health principles to address cardiovascular disease (CVD) through a combination of health care system and community-based interventions in Sonoma County, California. Central to this effort is the "It’s Up to Us" campaign, launched in 2017 by the United Way of the Wine Country and the Northern California Center for Well-Being, which aims to educate the community about CVD risk factors and empower individuals to take control of their health by checking their blood pressure. The Hearts of Sonoma County (HSC) initiative engages both clinical providers and community members to reduce CVD risk, involving activities like training community health workers for education and screenings, as well as media campaigns for heart disease prevention. The study highlights the demographics of Sonoma County, noting that about 31% of adults had been diagnosed with high blood pressure and 7% with heart disease from 2015 to 2017. |
| 4 | Corsino (2013), North Carolina, USA[55] | Focus group interviews | The Achieving Health for a Lifetime (AHL) project aims to reduce obesity by initially focusing on elementary school children, with plans to expand to include older children, parents, and older adults in the surrounding communities. Utilizing a social ecological framework, the project seeks to promote behavioral changes related to dietary habits and physical activity while addressing the community's concerns about obesity through education, services, and policy change. Community members identified barriers to weight management, such as financial issues, bad habits, and lack of motivation, while also highlighting the importance of social support and improvements in self-esteem as motivators for managing weight, particularly in the diverse population of Durham, where 41% are Black and 14.2% are Hispanic. |
| 5 | Curbach (2018), Germany[40] | Interviews, field notes, and focus group discussion | Empowering senior citizens (ages 60 to 75) in a rural community in Bavaria, Germany, to make healthier food choices and improve their food environment. It posits that participation in a nutrition group would be particularly appealing to seniors in this rural setting due to the scarcity of health-promoting programs compared to urban areas. The researchers believe that the weaker infrastructure and limited health initiatives in smaller rural communities contribute to greater disadvantage for these seniors relative to their urban counterparts. |
| 6 | Cutforth (2015), USA[41] | CBPR, content analysis | Improving physical education (PE) classes in 14 small, rural, low-income school districts in the San Luis Valley (SLV) of southern Colorado. The aim is to enhance the quality of PE instruction and increase the amount of moderate to vigorous physical activity during classes, with the goal of reducing obesity risk among students. The SLV serves approximately 7,500 students, of whom 70% qualify for free or reduced lunch and 50% are Hispanic, highlighting the community's socioeconomic challenges. |
| 7 | Davis (2017), Cuba[42] | Semi-structured interviews, meeting minutes, and local newspaper articles. | The Step Into Cuba Alliance, a community-university partnership aimed at promoting physical activity, particularly walking, in Cuba, New Mexico. This initiative includes the Village Interventions and Venues for Activity (VIVA) project, which serves as the evaluation component of the partnership. Key activities involve community-wide campaigns, improving access to physical activity locations, enhancing street design, fostering social support for physical activity, and implementing individually tailored programs, all within a rural, tri-ethnic community facing significant health disparities and socioeconomic challenges. |
| 8 | De Jong (2022), Netherlands[26] | Coordinated action checklist (CAC), composed network analysis, interviews, group sessions, minutes of the coalition meetings and reports of activities. | "Voorstad on the Move" (VoM), a community health promotion program in a low socioeconomic city district of Deventer, the Netherlands. The program aims to improve overall health and reduce health inequities within the community. By addressing the specific needs of the population, VoM seeks to foster healthier lifestyles and enhance well-being in the area. |
| 9 | De Jong (2023), Netherlands[56] | Evaluation sessions, interviews, group sessions, progress reports, minutes, activities database, photovoice study. | The "Voorstad on the Move" (VoM) coalition, a community health promotion program aimed at improving the perceived health of low socioeconomic status (SES) citizens in a deprived city district in the eastern Netherlands, home to 10,750 residents. The coalition consists of representatives from various local organizations, including the municipal health service, social support teams, welfare organizations, and sports services, all working collaboratively to support healthy behaviors and reduce health inequalities. VoM employs an open approach emphasizing citizen participation, intersectoral collaboration, and targeted changes in the social and physical environment. |
| 10 | De Marco (2014), USA[43] | CBPR | The Harvest of Hope project, a community-initiated pilot program aimed at addressing racial disparities in obesity and chronic disease risk among African-American youth and adults in a rural, low-resource county in North Carolina. Initiated by a Black church in partnership with public health researchers from the University of North Carolina (UNC) at Chapel Hill, the program employs gardening to provide nutrition education, increase physical activity, and improve access to fresh produce. This initiative responds to the county's significant challenges, including high rates of adult obesity, poor health, premature death, and unemployment. |
| 11 | Derose (2018), USA[44] | CBPR, semi-structured interviews, community meetings | The study focuses on the collaboration between religious congregations and public health organizations in South Los Angeles, a predominantly African-American and Latino area (95% as of 2017), to create a multi-ethnic, multi-denominational partnership aimed at addressing health disparities. The initiative emphasizes the development of a multi-level, multi-component church-based intervention, with obesity identified as the priority health issue. This area faces significant challenges, including higher proportions of Latino and African-American residents, lower socioeconomic status, poorer health outcomes (such as obesity and HIV), and is designated as both a medically underserved and health professional shortage area. |
| 12 | Dodgen (2020), USA[45] | CBPR | SHE Tribe (She’s Healthy and Empowered), a peer-led, social network-based lifestyle intervention targeting underserved women. This project builds on two prior initiatives: one research-focused project aimed at implementing and testing a culturally relevant, faith-based Diabetes Prevention Program among African American women in churches, and another outreach-focused project that emphasized community capacity building for sustainable obesity prevention activities in underserved neighborhoods. The SHE Tribe intervention seeks to empower women through culturally relevant approaches and community engagement. |
| 13 | English (2023), Australia[46] | community-based research, meeting minutes, workshop review feedback and program debrief notes. | Key learnings from a collaborative research experience involving both Aboriginal and Torres Strait Islander and non-Indigenous stakeholders in co-designing a physical activity and psychosocial health program for Aboriginal and Torres Strait Islander girls. The Tidda Talk program aims to enhance the social and emotional well-being and physical activity behaviors of adolescent females aged 11 to 16 through weekly group-based psychosocial education and physical activity sessions. The KARI Foundation, an Aboriginal non-profit organization, is dedicated to empowering communities through education and programs that support youth and families, with six program pilots planned in the greater Sydney region. |
| 14 | Fialkowski (2013), US Affiliated Pacific Island, Hawaii and Alaska[47] | Interviews, community meetings and community feedback meetings | Addressing childhood obesity among remote, underserved minority populations in the US Affiliated Pacific Islands (USAPI), Hawaii (HI), and Alaska (AK). This region is characterized by its vast and isolated geography, covering over one million square miles in the Pacific Ocean, which is larger than the land area of the contiguous United States. The remoteness and diversity of the area contribute to a variety of small, widely dispersed cultures, presenting unique challenges and opportunities for tackling childhood obesity. |
| 15 | García-Rivera (2017), Puerto Rico[48] | Survey, in-depth interviews, focus groups and intervention mapping. | The "Salud para Piñones" participatory research project, which aimed to integrate elementary school children (grades 4-6) in the community of Torrecilla Alta (Piñones) into an intervention promoting fruit and vegetable consumption from 2011 to 2012. From 2012 to 2014, the project developed a community health worker model and employed intervention mapping and mixed methods analysis to address chronic disease self-management. Key health priorities identified through community engagement included the lack of emergency and preventive services, high rates of hypertension and diabetes among adults, and high asthma prevalence in children, all within a low-income, high-risk community facing significant health disparities and social challenges in the Loíza municipality of Puerto Rico. |
| 16 | Hargreaves (2016), USA[27] | Interviews, analysis of team measures and documents, observations of learning sessions, and analysis of team surveys and other feedback. | A strategic initiative that aims to (1) build multisector teams to enhance collaborative capacity, (2) provide services to support health behavior changes for children and their families, and (3) promote sustainable social and environmental policy changes at both organizational and community levels. The initiative seeks to foster partnerships between primary care, public health, and community-based organizations to identify sustainable methods for promoting healthy weight and reducing health disparities across the United States. The community-based teams involved in the project were diverse, varying in community setting (urban vs. rural), team size, prior experience, and representation from different sectors (primary care, public health, community), though some differences were noted between the groups across different phases of the initiative. |
| 17 | Heo (2018), South Korea[57] | Face-to-face, semi-structured interviews network analysis | Residents of Single Room Occupancies (SROs) in Seoul, primarily older males aged 50 and above who live alone, with 40% having experienced homelessness in the past. These residents face significant challenges, including deprivation, isolation, community fragmentation, declining mental health, and alcohol abuse. The study aims to explore how various community actors currently collaborate and share information to promote health, their attitudes towards future collaboration, and perceived barriers to working together. It seeks to enhance understanding of community relationships and develop a practical health governance model based on intersectoral collaboration to reduce health inequities. Additionally, the community-based organization (CBO) involved has established self-support groups focusing on managing chronic conditions, crime prevention, street cleaning, and community gardening to promote overall health in the SRO-Z area, where many residents have precarious housing conditions. |
| 18 | Hilgendorf (2016), Wisconsin, USA[58] | Process evaluation data and reflections of lead community partners. | The Wisconsin Obesity Prevention Initiative, which combines coalition action and community organizing to address obesity prevention. Coalition action involves diverse community representatives collaborating for collective impact, while community organizing engages residents in sustained social action to tackle shared local concerns. The initiative operates in Marathon County, a primarily rural area with a population of 134,063, predominantly white (approximately 90%), but also includes Southeast Asian and growing Latino/Hispanic communities. Additionally, it addresses health issues in Menominee County, home to the Menominee Nation, where nearly 90% of the population is Indigenous and faces high rates of overweight, obesity, and chronic diseases. |
| 19 | Holt (2023),  Denmark[28] | Interviews, focus groups, and observations. | The Fit Kids Play initiative aims to improve the health and well-being of children with obesity by involving them in regular team sports organized by community sports clubs and funded by local authorities. The steering committee consisted of a doctor from a children's obesity clinic, a national NGO supporting children from disadvantaged backgrounds, and a regional non-profit sports association. The partnership also collaborates with local sports clubs and municipalities to secure funding and recruitment pathways for the target group. The municipalities, responsible for welfare services in Denmark, play a key role in supporting the initiative. |
| 20 | Igel (2018), Germany[29] | According to the importance of learning through ‘anecdotal evidence’ (Poland et al., 2009) and reflexivity (Finlay, 2002), our experiences in the field of community-based participatory health promotion (as an example of ‘multiple disciplinary’ work (Choi and Pak, 2006) are described and analysed. Drawing on minutes from numerous meetings, email conversations and personal research diary entries of the staff. | The challenges encountered in inter- and transdisciplinary work aimed at improving child health and preventing obesity in a socially disadvantaged community in Grünau, Leipzig. The research examines the positions, perceptions, and interactions of researchers with each other and community members. The initiative targets changes in the "obesogenic environment" by addressing physical and social characteristics across various settings, such as schools, childcare centers, and neighborhoods. Key interventions include raising awareness among families and caregivers, strengthening community ties, modifying green spaces and playgrounds to encourage physical activity, and enhancing cooperation among different organizations. |
| 21 | Jenkins (2020), Australia[59] | Focus group, semi-structured interviews. | Children aged 7-12 years and the Sustainable Eating Activity Change Portland (SEA Change Portland) initiative. The program aims to achieve lasting changes in weight status by implementing community-generated and led modifications to the environments where children live, learn, and play. Its objectives include promoting healthy eating in schools, increasing physical activity and active transport, enhancing water consumption, and reducing unhealthy local marketing, sponsorship, and fundraising. The initiative is based in the regional seaside town of Portland, Victoria, Australia, which has a population of approximately 9,700 residents and is located 360 km from Melbourne. |
| 22 | Johnson-Shelton (2015), USA[60] | CBPR, written record of discussions, decisions, procedural developments, and accomplishments in the project, survey, neighborhood built environment assessment, and food environment audits. | School children within the Communities and Schools Together (CAST) project, which targets childhood obesity prevention through place-based health promotion. The project defines four main communities: (1) the school district, including administrators, teachers, and staff; (2) the parents and families of seven elementary schools; (3) community NGO groups; and (4) the research and academic community. It aims to leverage the influence of these local communities on child physical activity, food environments, and nutrition within a school district located in the southern Willamette Valley of Oregon, USA. |
| 23 | Jones (2017), Australia[30] | Focus groups and interviews | Children aged 0-18 years and the OPAL (Obesity Prevention and Lifestyle) program, which is a systems-wide initiative for childhood obesity prevention. OPAL utilizes ecological systems theory along with community development and social marketing strategies to effect changes in eating and activity behaviors and their environments. The program operates in six communities across South Australia, including four metropolitan and two rural areas. |
| 24 | Kennedy (2019), Canada[31] | SCOPE’s Partnership Tracking Tool (PTT), the Public Health Agency of Canada’s (PHAC) Community Capacity Building Tool (CCBT), and semi-structured interviews. | The Live 5-2-1-0 initiative, a multi-sectoral, multi-component program aimed at preventing childhood obesity. It involves collaboration among a diverse group of community stakeholders, including local government, health, education, and businesses. The initiative targets children and is implemented in various communities throughout British Columbia (BC), Canada. |
| 25 | Korn (2018), Multisite - USA, Australia and Europe[49] | Survey and interview | Community-based childhood obesity prevention interventions that involve coalitions in their design, implementation, and evaluation. Study aim is understanding the processes and dynamics of coalition engagement in these interventions, using the Community-Based Participatory Research (CBPR) Conceptual Model. |
| 26 | Lachance (2018), USA[32] | Document analysis. | Food and Fitness (F&F) community partnerships aimed at enhancing access to locally grown food and safe spaces for physical activity. It targets children and families in racially, ethnically, and geographically diverse communities facing inequities across the United States. |
| 27 | Lolacono Merves (2015),  USA[50] | CBPR | The Bronx Youth as Partners in Community-Based Participatory Research (BYAP), an eight-year partnership aimed at preventing and reducing mental health disparities among Latino and Black non-Latino adolescents in the Bronx, New York. Funded by the National Institute on Minority Health and Health Disparities, the initiative included a steering group called "Albert's Leaders of Tomorrow" (ALOT), composed of 14-20 adolescents and 3-6 adult researchers and clinicians with expertise in adolescent health. Participants, aged 13 to 19 and residing in the Bronx, were recruited from local schools and agencies. |
| 28 | Majee (2015), USA[51] | In-depth interviews and document review. | The study focuses on a university-community partnership between the University of Missouri’s Healthy Communities Initiative (HCI) and Sedalia’s Blue Ribbon Health and Wellness Planning Team (BRHWPT) aimed at enhancing community health. BRHWPT consists of sub-coalitions targeting five thematic areas: clean air, adolescent health, healthy living, breastfeeding, and healthcare professional recruitment and retention. Sedalia, Missouri, has a population of 21,492, characterized by a comparatively low median household income, high rates of low-paying manufacturing jobs, and 17.2% of residents living below the poverty line, surpassing the state average. |
| 29 | Matheson (2019), New Zealand[33] | Interviews, online stakeholder survey, document analysis. | The focus of the study is on Healthy Families NZ, an initiative designed to enhance the health prevention system by promoting healthy food choices, physical activity, and smoke-free, alcohol-free environments. It employs a community-based approach, with locally based Lead Providers overseeing implementation and chairing Local Strategic Leadership Groups that include diverse stakeholders from health, government, Māori, business, and education sectors. The initiative is guided by five building blocks for a robust prevention system and operates in ten geographic communities in New Zealand, primarily targeting areas with high rates of preventable chronic disease risk factors and socio-economic deprivation, encompassing both urban and rural settings. |
| 30 | McEvoy (2019), Ireland[61] | Documentary analysis, semi-structured interviews and focus groups. | The focus of the study is the Joint Community Participation in Primary Care Initiative (JI), which includes 19 demonstration projects aimed at enhancing the participation of excluded communities in primary health care (PHC) across disadvantaged areas in Ireland. Activities range from health promotion interventions, such as drug and alcohol awareness and suicide prevention programs, to community needs assessments and representation on Primary Care Teams (PCTs). Two of the projects specifically target marginalized groups, including travellers and the minority ethnic community, within both rural and urban disadvantaged settings. |
| 31 | McNeish (2019), USA[34] | Document review, semi-structured individual and/or group interviews, direct observation of activities and meetings. | The Making Connections Initiative (MCI) is a national community-based intervention in the U.S., funded by the Movember Foundation and coordinated by the Prevention Institute, aimed at improving the mental health and well-being of men and boys, particularly those at higher risk, such as men and boys of color and military veterans. The initiative involves activities like needs assessments, partnerships, and visioning to develop actionable plans for community change. A notable challenge is the significant variation in program scope and population focus among the funded sites, complicating the understanding of community-level implementation. |
| 32 | Middleton (2013), UK[35] | Semi-structured interviews and focus groups. | The community-based obesity prevention program operates across a network of organizations, including local authorities, businesses, charities, and health professionals, to collaboratively design and deliver interventions aimed at changing nutrition and physical activity behaviors. It includes 32 specific interventions targeting the main determinants of obesity in various settings such as schools, children's centers, workplaces, and community facilities. Located in a Care Trust area in the North East of England, the program serves a population of approximately 160,000, with many demographic segments ranking among the top 10% of the most deprived areas in the country. |
| 33 | Nelson (2013), USA[62] | Semi-structured interviews | The "Eat Smart, Move More" (ESMM) projects focus on increasing physical activity among young people aged 9 to 14 years through initiatives in schools and after-school programs across 20 counties in North Carolina. The study explores the characteristics of strong partnerships from the perspective of community project coordinators involved in promoting these physical activity programs. |
| 34 | Ochieng (2021), UK[52] | Focus group discussions, co-creation activity and workshops | A co-creation intervention aimed at promoting healthy weight maintenance in early childhood, specifically targeting Black African migrant children. It emphasizes partnership work with these participants to develop culturally sensitive resources and frameworks. The need for a multifaceted approach involving individuals, families, communities, local businesses, and broader societal changes to effectively address childhood obesity and support healthy dietary practices. |
| 35 | Ottesen (2024),  Sweden[63] | Semi-structured Interviews | Exploring stakeholder perspectives on co-designing health promotion efforts with citizens to prevent cardiometabolic diseases (CMD)—such as type 2 diabetes, stroke, and heart disease—in socioeconomically disadvantaged neighborhoods in the Uppsala region, Sweden. |
| 36 | Powell (2019), Australia[64] | Documents review and semi-structured interviews. | "Our Healthy Clarence" is a community-driven mental health and wellbeing initiative in Northern New South Wales, launched in response to a local cluster of suicides. The initiative has five main objectives: improving access to treatment and crisis care for those at risk of self-harm, enhancing community and worker responses to self-harm risks, ensuring mental health programs are available in schools, increasing community awareness of mental health resources, and strengthening community connections. Targeting individuals at risk of self-harm and the broader community, the program aims to provide early support and prevention strategies in the socioeconomically disadvantaged Clarence Valley Local Government Area, which has a population of 51,570 residents. |
| 37 | Pratt (2017), USA[36] | Semi-structured interviews. | A collaborative initiative involving practice-based research networks (PBRNs) from four states aimed to explore the integration of primary care (PC) and public health (PH) practices. The project focused on the continuum of integration proposed by the Institute of Medicine, which includes stages like mutual awareness, cooperation, collaboration, and partnership. It examined the dimensions of integration and identified factors that facilitate or hinder this process from a local perspective. The study involved PC and PH professionals across 20 local jurisdictions in Colorado, Minnesota, Washington, and Wisconsin. |
| 38 | Quinn (2018), USA[53] | Survey and interviews. | The Food & Fitness Initiative focused on creating community-led approaches to improve access to locally grown food and healthy places for physical activity, particularly for children and families in underserved areas across the U.S. The initiative aimed to foster sustainable changes in policies, environmental infrastructures, and systems. Youth participated in partnership-established advisory boards, gaining experience through internships and volunteer work to enhance access to healthy food. Key accomplishments included changes in local food systems, such as supportive zoning policies for urban agriculture, increased farmer’s markets and community gardens, and expanded healthy food options in schools and stores. The initiative also improved active living environments with new greenways, parks, and pathways. Overall, the emphasis was on building capacity within communities to ensure that efforts are community-driven and sustained. |
| 39 | Rämgård (2022), Sweden[65] | Notes from monthly meetings, interviews and focus groups. | The Lindängen initiative is a community-based participatory research program aimed at promoting equitable health in a low-income neighborhood in Malmö, Sweden. It involves lay health promoters (LHPs) to develop an innovative model for community health promotion. Lindängen has a population of about 7,800, with 80% being first- and second-generation immigrants. The area faces significant challenges, including an unemployment rate of approximately 45%, compared to the city's average of 15%, and a notably higher incidence of ill health days among residents aged 20–64, which is 50% above the city average. |
| 40 | Sjögren Forss (2021), Sweden[37] | Community Based Participatory Research, and interviews. | The Collaborative Innovations for Health Promotion program is a community-based participatory research (CBPR) initiative in Malmö, Sweden, focused on improving health through participatory and collaborative strategies. Targeting the Lindängen area, which has about 7,600 residents—75% of whom are first- and second-generation migrants—the program addresses key health issues identified by the community, including lack of physical activity, poor oral health, and mental health problems. The area faces significant challenges, with approximately 50% unemployment for both men and women, high crime rates, and substantial health inequalities. |
| 41 | Storm (2015), Netherlands[14] | Documentary analysis, (group) interviews, questionnaires, and observations. | A stepwise approach using two key tools—a district health profile and policy dialogue—to develop integrated district plans and enhance collaboration in seven neighborhoods of Noord-Brabant, Netherlands. A core team for each neighborhood, comprising an epidemiologist and policy advisors from regional public health services and primary care support, supervised the process. Key themes addressed included obesity, social cohesion, and loneliness among the elderly. The neighborhoods were chosen based on existing relationships, collaborative openness, geographic diversity, and municipality size, including two medium-sized and five small municipalities. |
| 42 | Toft (2018), Denmark[38] | Quasi-experimental study.  Intervention development: development of program theory with inspiration from Realist Evaluation; participatory approaches; action research approach. | Promoting healthier dietary habits and physical activity among families with children aged 3 to 8 years in three local communities (Nexø, Hasle, and Allinge-Sandvig) in the Regional Municipality of Bornholm, Denmark. It aims to mobilize local resources, strengthen social networks, and reduce social inequality. The intervention communities are compared to three equivalent control communities (Asnæs, Højby, and Egebjerg) in Odsherred Municipality. Bornholm has a population of approximately 42,000, while Odsherred has about 32,500 inhabitants. |
| 43 | Vargas (2023),  Australia[54] | Survey and focus groups | The focus of the *RESPOND* study is to promote children's health and prevent childhood obesity in rural and regional areas of northeast Victoria, Australia. It uses a community-led, whole-of-systems approach, engaging local stakeholders through group model building (GMB) workshops to identify the key causes of unhealthy weight in children. Based on these insights, the community co-designs and prioritizes locally relevant actions aimed at improving children's physical activity, food environments, and health behaviors. These actions are implemented within the community to address the complex factors contributing to childhood obesity. The study is conducted in Mansfield, a small rural town with a population of 4,787, including 927 children aged 0–14 years. |
| 44 | Vermeer (2013), Netherlands[66] | semi-structured questionnaire contained open-ended questions | Five community health programs in deprived neighborhoods as part of the "Uw buurt gezond!" ("A Healthy Neighborhood") project in Limburg, Netherlands. The programs aim to reduce socioeconomic health inequalities and address health issues, particularly related to cardiovascular diseases and mental/social well-being. Interventions include establishing exercise facilities, school policies on birthday treats, and cooking courses for adults. The neighborhoods, which have populations ranging from 2,500 to 5,000, are located in a former mining region characterized by high unemployment and various social and health challenges. |
